# A novel 3D-printed head phantom with anatomically realistic geometry and continuously varying skull resistivity distribution for electrical impedance tomography

**DOI:** 10.1038/s41598-017-05006-8

**Published:** 2017-07-04

**Authors:** Jie Zhang, Bin Yang, Haoting Li, Feng Fu, Xuetao Shi, Xiuzhen Dong, Meng Dai

**Affiliations:** Faculty of Biomedical Engineering, Fourth Military Medical University, 169 West Changle Road, Xi’an, 710032 China

## Abstract

Phantom experiments are an important step for testing during the development of new hardware or imaging algorithms for head electrical impedance tomography (EIT) studies. However, due to the sophisticated anatomical geometry and complex resistivity distribution of the human head, constructing an accurate phantom for EIT research remains challenging, especially for skull modelling. In this paper, we designed and fabricated a novel head phantom with anatomically realistic geometry and continuously varying skull resistivity distribution based on 3D printing techniques. The skull model was constructed by simultaneously printing the distinct layers inside the skull with resistivity-controllable printing materials. The entire phantom was composed of saline skin, a 3D-printed skull, saline cerebrospinal fluid (CSF) and 3D-printed brain parenchyma. The validation results demonstrated that the resistivity of the phantom was in good agreement with that of human tissue and was stable over time, and the new phantom performed well in EIT imaging. This paper provides a standardized, efficient and reproducible method for the construction of a head phantom for EIT that could be easily adapted to other conditions for manufacturing head phantoms for brain function research, such as transcranial direct current stimulation (TDCS) and electroencephalography (EEG).

## Introduction

Electrical impedance tomography (EIT) seeks to reconstruct the changes in impedance distribution within tissues caused by related physiological and pathological activities; using the data from injecting a set of currents into the body through surface electrodes and measuring the boundary voltages^1^. EIT has promising value in applications detecting or monitoring cerebral haemorrhage^2^, cerebral ischaemia^3^, brain oedema^4^ and other critical diseases of the head^1, 5^ given its non-invasive nature, lack of radiation, functional imaging, and ability for real-time monitoring.

In general, in the process of brain EIT studies, phantom experiments are an important step for testing during the development of new hardware or imaging algorithms. By bridging between computer-based simulations and clinical measurements, studies in phantoms could systematically investigate the performance of the developed data-acquisition system, reconstruction algorithms, and imaging software^6–8^ and subsequently provide reasonable information for further optimization or experiments. Therefore, to ensure the accuracy and reliability of the results of phantom experiments, there is a clear need for a realistic phantom that mimics the real geometry and resistivity distribution of the human head as closely as possible.

However, due to the sophisticated anatomical geometry and complex resistivity distribution of the human head, constructing an accurate phantom for EIT research remains significantly challenging, especially for skull modelling. As demonstrated by previous studies, the skull can be anatomically divided into distinct layers, including the top and bottom layers of compact bone with high resistivity and the middle layer of diploe with relatively low resistivity^9, 10^. The resistivity of a specific skull section was thus determined by the percentage on thickness of diploe (PTD) and the resistivity distribution of the entire skull presented as spatially inhomogeneous owing to variations in PTD throughout the skull^11^. Therefore, how to properly model the inhomogeneity is a critical issue for accurately building an EIT head phantom.

To date, numerous efforts have been made to create a head phantom for the brain applications of EIT. In 2004, the University College London (UCL) group established a simple spherical skull phantom with a realistic resistivity of 83.3 Ω·m by casting plaster of Paris, but its shape significantly differed from the real head geometry^12^. The group also produced a head tank by employing a real human skull and a marrow or giant zucchini imitating the skin. The tank was successfully used in time difference and frequency difference EIT research, but the tank using dead skull tissue tended to overestimate the resistivity^8^. In 2012, Sperandio *et al*. constructed a four-shell diffusion phantom of the head for EIT using agar gel thickened saline of different concentrations, wherein they used a volume conductive film between shells to prevent ion transfer. The phantom included four layers for skin, skull, cerebrospinal fluid (CSF) and brain parenchyma and demonstrated accurate modelling of the resistivity of skull. However, the model was hemispherical and ignored the anatomical structure of head, which might lead to unreal current density distribution^13^. In 2014, our group fabricated a plaster phantom with anatomically realistic skull shape and spatially varying skull resistivity distribution. In this phantom, after obtaining a 3D skull geometry from computed tomography (CT) reconstruction, we anatomically divided the skull model into eight separated sections (one frontal bone, two sphenoid wing bones, two temporal bones, two parietal bones and one occipital bone), wherein each section had a specific resistivity value that was replicated by changing the ratio of dental-grade plaster to distilled water^14^. Although the resistivity distribution of this skull phantom represented to be inhomogeneous among sections, the resistivity was constant across each section. The phantom could not produce a continuously varying skull resistivity distribution because it was unable to model the multi-layer structure of skull.

As an emerging manufacturing technology, 3D printing has been studied and applied in numerous different areas^15, 16^. In the field of biomedical engineering, exploring methods to accurately fabricate the human tissue model using 3D printing has received considerable attention^17, 18^, and one of the areas involves the construction of physical models for medical imaging experiment. Iida *et al*. proposed a 3D printed head physical model for positron emission computed tomography (PET)/single-photon emission computed tomography (SPECT) to evaluate imaging system performance^19^. Gatto *et al*. proposed a head model based on 3D printing and applied it to ultrasound imaging^20^, wherein the main focus of the study involved the ultrasound properties of the model. Regarding brain EIT, the ideal method to correctly replicate the complex resistivity distribution of the human head is an important prerequisite for constructing the EIT head phantom using 3D printing. In 2015, the UCL group proposed a novel 3D-printing-based method of creating EIT head phantoms with realistic geometry and spatially variable skull resistivity, in which a series of perforations were cut into the surface of the skull model. In addition, the exact resistivity could be controlled by the ratio of saline within the holes to solid insulating plastic or by altering the density or diameter of these holes^21, 22^. However, their 3D printed phantom did not model the trilayer structure of the skull, which was supposed to more accurately represent the resistivity distribution of skull^10^. In addition, similar to most previous studies, the researchers only used saline to replicate the resistivity distribution of brain parenchyma for simplification, instead of creating a model with structures representing superficial sulci and gyri.

Alternatively, in this study, we first attempted to directly control the resistivity of 3D printing materials by altering the mixture proportion of candidate conductive composites. Afterwards, based on 3D printing techniques, a novel head phantom with anatomically realistic geometry and continuously varying skull resistivity was created, wherein we improved the modelling of the inhomogeneous distribution of skull resistivity by simultaneously printing the distinct layers inside using the proposed resistivity-controllable printing materials. In addition, the geometry of brain parenchyma with superficial sulci and gyri was also incorporated. The phantom was completely composed of saline skin, a 3D-printed skull, saline CSF and 3D-printed brain parenchyma. Its performance was subsequently validated in terms of the accuracy of resistivity distribution and geometry.

## Results

### The relationship between the resistivity and proportions of ABS/CB_10%_ for 3D-printed samples

**Figure 1:**
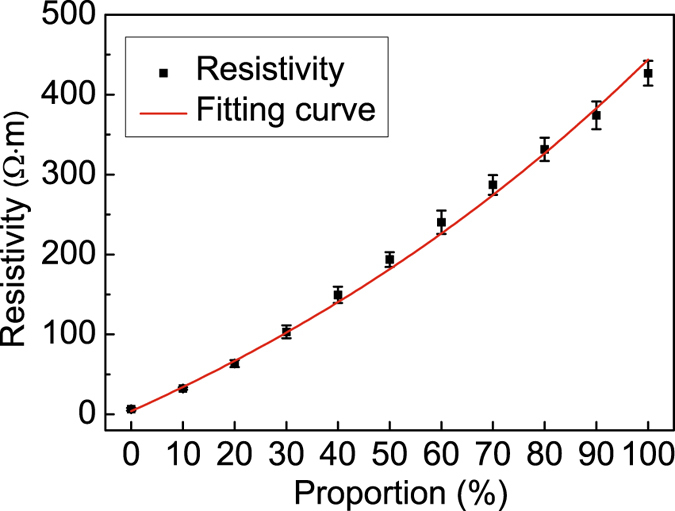
The resistivities of 3D-printed samples after mixing ABS/CB_10%_ and ABS/CB_20%_ with different proportions (mean ± SD, n = 6) and the fitting curve between the resistivity and the proportion of ABS/CB_10%_ (Frequency = 1 kHz).

Two types of acrylonitrile butadiene styrene (ABS)/carbon black (CB) particles with volume fractions of 10% and 20% CB (referred to as ABS/CB_10%_ and ABS/CB_20%_, respectively) were selected given that their resistivities cover the range of skull and brain parenchyma. The particles were fabricated into filament with a diameter of approximately 1.75 mm using a single screw extruder in preparation for 3D printing (Fig. S1)^23^. Cubic samples (20 mm x 20 mm x 20 mm) were printed using the prepared filaments for resistivity measurement. The resistivities of samples were measured by an impedance analyser and the four-electrode method (Fig. S1). The resistivities of two ABS/CB composites appeared relatively stable over the frequency and time domains (Fig. S2). To control the resistivity of 3D printing materials, the resistivity of the mixture of ABS/CB_10%_ and ABS/CB_20%_ with different proportions at the frequency of 1 kHz was investigated. The resistivities increased with the proportion of ABS/CB_10%_ for cubic samples as shown in Fig. 1. The exponential function was used to fit the resistivity to the proportion of ABS/CB_10%_ as the relationship between resistivity and content of conductive agent was always an exponential function^24^. The results are presented in Fig. 1, and the regression equation can be expressed as

1 $$y=373.35{e}^{(x/128.45)}-369.36$$

where *y* is the resistivity of the mixed material of cubic samples and x is the proportion of ABS/CB_10%_.

The correlation coefficient of the fitting function is 0.995. Thus, the 3D printing materials with the resistivity consistent with skull and brain parenchyma could be accurately obtained using the equation.

### Skull model with inhomogeneous resistivity distribution

**Figure 2:**
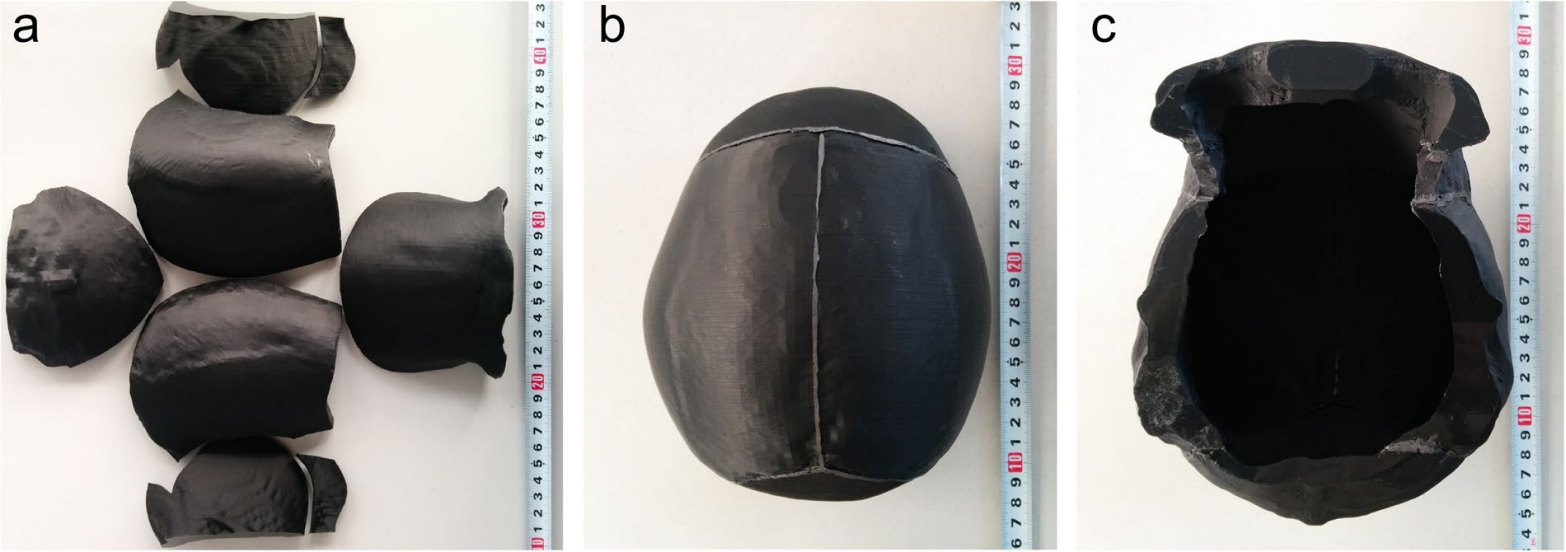
Separated skull model and completed skull model. (**a**) Separated skull model including eight sections; (**b** and **c**) completed skull model after splicing.

First, we obtained the computer-aided design (CAD) model of the skull by medical imaging and 3D reconstruction (Fig. S3). Second, the skull model was segmented in accordance with the suture, and the segmented skull model was sliced into a structure similar to that of the skull anatomy. Thus, the cross section was divided into a trilayer structure containing the diploe and the compact bone or single-layer compact bone structure according to the thickness (Fig. S3b and S3c). Finally, the eight skull sections with distinct layer structures were printed using materials whose resistivity was similar to the compact bone or diploe (Fig. 2a). The models were then spliced using conductive epoxy adhesive to obtain the entire skull model (Fig. 2b and c).

To evaluate the resistivity distribution of the proposed skull phantom, we made comparison between the previous study on the resistivity of live human skulls and the present 3D-printed skull phantom. Tang *et al*.^11^ from our group performed the most recent study on skull resistivity. They classified the skull into six categories according to different structures of the skull samples: four categories without suture, including standard trilayer skull (79.43 ± 17.52 Ω·m), quasi-trilayer skull (144.71 ± 30.61 Ω·m), standard compact skull (265.46 ± 53.74 Ω·m), and quasi-compact skull (198.24 ± 32.32 Ω·m); two categories with suture, including dentate suture skull (57.82 ± 17.78 Ω·m) and squamous suture skull (127.47 ± 41.20 Ω·m). Without suture, the resistivity of human skull varied with the thickness or PTD. The absence of diploe appeared to increase skull resistivity.

**Figure 3:**
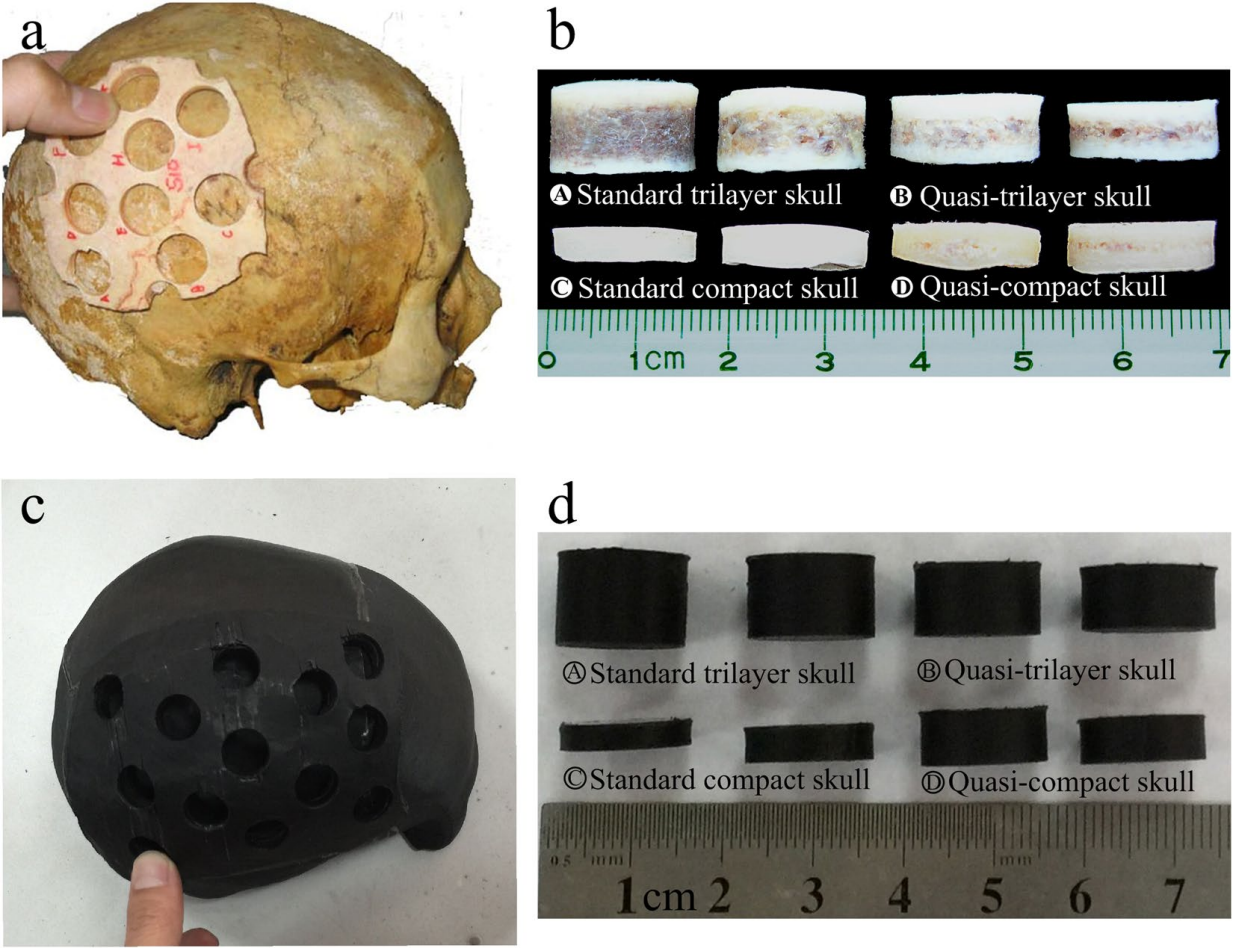
Live skull samples and 3D-printed skull samples^11^. (**a**) The live skull sample; (**b**) four categories of skull sample: standard trilayer skull, quasi-trilayer skull, standard compact skull, and quasi-compact skull; (**c**) 3D-printed skull phantom sample; (**d**) four categories of 3D-printed phantom sample, which are the same those noted for the live human skull.

**Figure 4:**
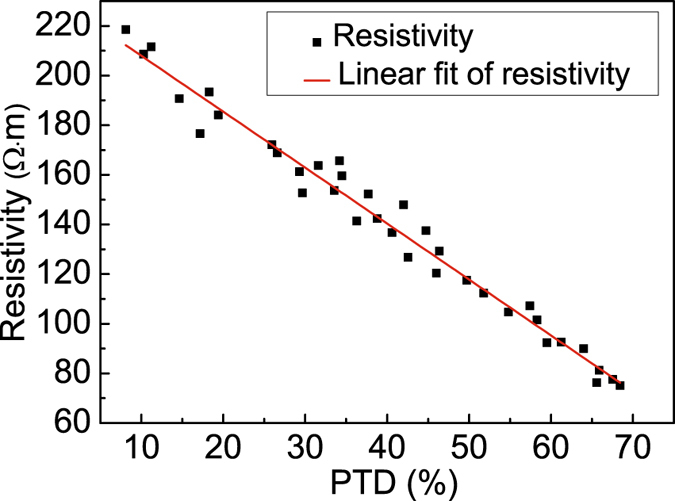
Relationship between PTD of 3D-printed trilayer skull and resistivity at the frequency of 1 kHz (correlation coefficient: r = −0.977).

To compare the resistivity distribution of the 3D-printed skull with that of live human skull, we measured 48 plugs excised from 3D-printed skull sections, as previously performed on live human skull^11^ (Fig. 3). Among the four categories of 3D-printed skull models without suture, the resistivity was shown in Table 1 (Mean ± SD, n = 12). The two-sample t test was applied to test the difference in resistivity for each skull category, and Table 1 reveals no significant difference in resistivity between the proposed skull phantom and the live human skull (p > 0.05). Moreover, given that an inverse relationship existed between PTD of trilayer skull and resistivity at the frequency of 1 kHz^11^, we fit the relationship between PTD of 3D-printed model and resistivity, and the relationship is consistent with that of human skull (Fig. 4). The comparison results indicated the resistivity distribution of 3D-printed skull was similar to that of live human skull.

**Table 1:** Resistivity comparison between 3D printed skull phantom and live human skull (Frequency = 1 kHz).

Characteristic	3D printed model (Ω·m)	Human skull (Ω·m)	p
Standard compact skull	236.93 ± 47.35	265.46 ± 53.74	0.080
Quasi-compact skull	183.86 ± 27.28	198.24 ± 32.32	0.129
Quasi-trilayer skull	141.07 ± 17.59	144.71 ± 30.61	0.542
Standard trilayer skull	92.56 ± 21.30	79.43 ± 17.52	0.065

The data were presented as mean ± SD (n = 12), and the two-sample t test was applied to test the difference.

Regarding the geometric accuracy of the 3D-printed skull model, we defined and measured the linear distance of 15 landmarks for the left parietal bone and the entire skull model (Fig. S4). Table 2 presents the mean and percentage of the absolute differences for the linear measurements of those landmarks. All dimensional errors were less than 2%.

**Table 2:** Mean and percentage of the absolute differences for the linear measurements.

Landmark name	CAD model (mm)	3D printed model (mm)	Absolute difference	
			mm	%
AB	77.78	76.91	1.98	1.12
BC	99.22	101.01	0.93	1.80
CD	100.17	99.24	1.79	0.93
AD	121.73	119.75	0.87	1.63
T1	9.36	9.23	0.13	1.39
T2	6.32	6.29	0.13	0.47
T3	10.58	10.37	0.03	1.98
T4	7.85	7.72	0.21	1.66
H1	28.23	28.52	0.29	1.03
H2	14.91	14.71	0.2	1.34
H3	51.32	50.58	0.74	1.44
H4	12.25	12.28	0.03	0.24
SL	182.93	185.46	2.53	1.38
SW	147.21	149.62	2.41	1.64
SH	117.9	118.38	0.48	0.41

### The establishment of the experiment platform and resistivity stability verification

**Figure 5:**
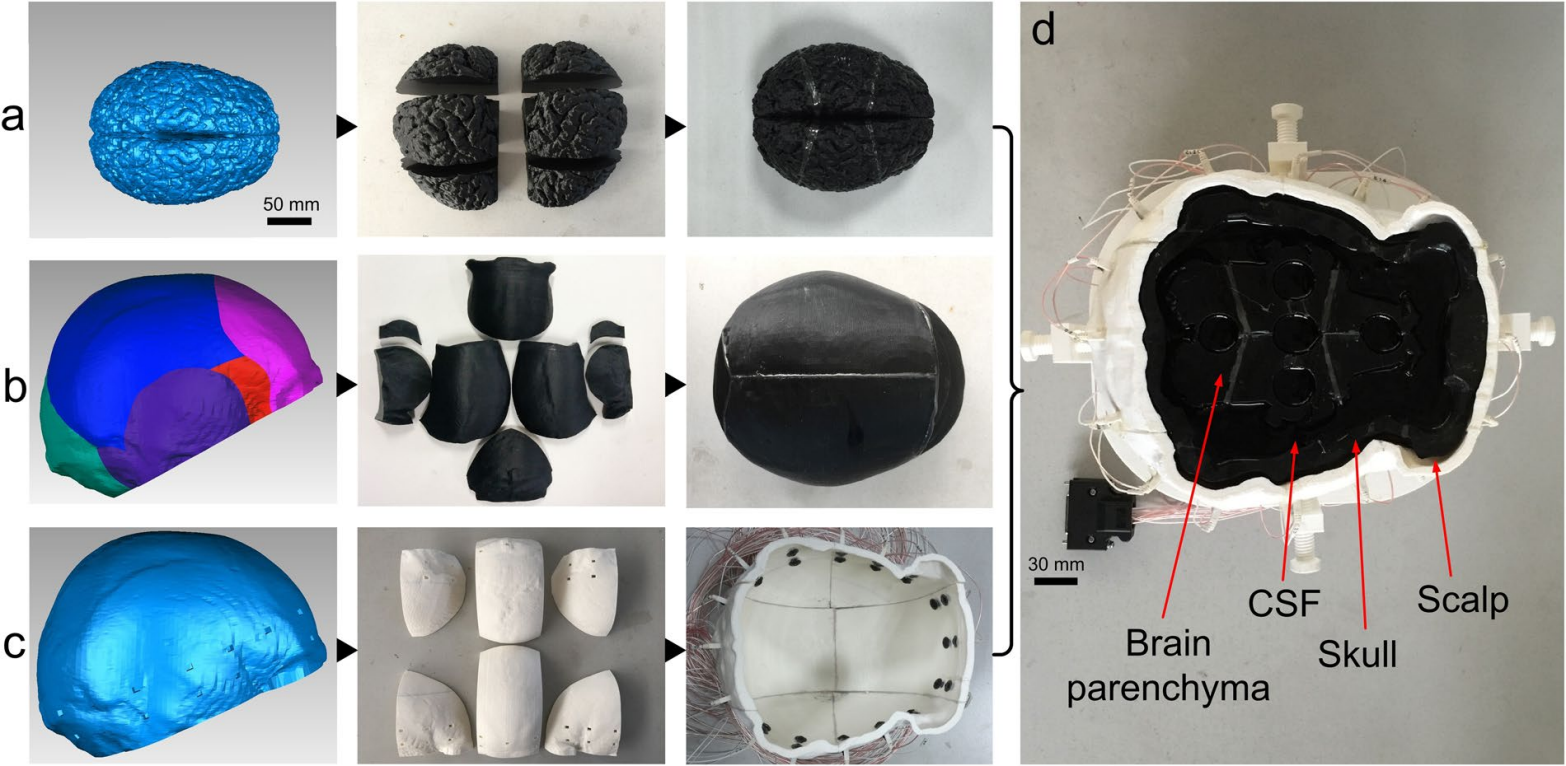
The fabrication of the head phantom using 3D printing techniques, including the CAD model, detached physical model, and the spliced physical model. (**a**) The brain parenchyma model; (**b**) the skull model; (**c**) the outer container model including two layers of Ag/AgCl electrodes; (**d**) the completed head phantom including the four-layer structure.

A new head phantom with a four-layer structure was fabricated based on the 3D-printed skull model (Fig. 5). The four-layer structure included scalp, skull, CSF and brain parenchyma. In the process of creating the head phantom, the brain parenchyma model was successfully fabricated using 3D printing, and its resistivity was close to that of the head tissue by regulating the 3D printing material. The model also replicated the superficial sulci and gyri of brain parenchyma (Fig. 5a). Moreover, in addition to brain parenchyma and skull (Fig. 5b), an outer container used for fixing electrodes was also fabricated with the non-conductive ABS by 3D printing (Fig. 5c).

When assembling the head phantom from different detached tissue models to integrate the four-layer structure, three small cylinders were attached to the inner surface of the skull to fix the parenchyma model, and a NaCl solution (concentration 0.9%, resistivity 0.56 Ω·m) with resistivity similar to the CSF (Table S1) was added to the space between the brain parenchyma and the skull to simulate CSF. Afterward, the skull was fixed in the outer container, and a NaCl solution with resistivity of 2.27 Ω·m, which is similar to that of scalp, was added to the space between skull and outer container to simulate the scalp. To prevent evaporation of the solutions, the head phantom was sealed using a thin film. In addition, two layers of Ag/AgCl electrodes were attached to the inner surface of the outer container to subsequently connect a brain EIT device.

**Figure 6:**
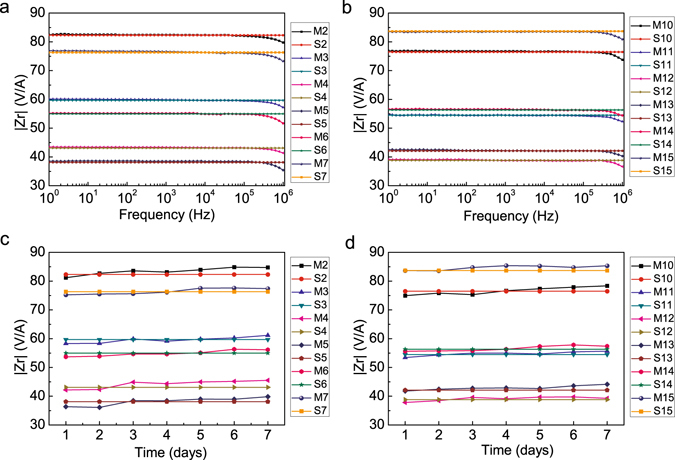
Comparison of the transimpedance measurements and simulation results. (**a** and **b**) Comparison of transimpedances at 1 Hz – 1 MHz; (**c** and **d**) transimpedance stability over time for 7 days (Frequency = 1 kHz). M: Measurement; S: Simulation.

In addition, the stability of the phantom resistivity was investigated by measuring the transimpedance and comparing these values with the simulation results as reported previously^13, 14^. The transimpedance measurements from different pairs of adjacent electrodes and simulation results were generally consistent, as shown in Fig. 6a and b. Specifically, the transimpedance between different pairs of adjacent electrodes was consistent with the simulation results at low frequencies (the deviation is less than 0.5%). After the frequency increased to 3 × 10^5^ Hz, the measured transimpedance decreased slightly. Moreover, considering the stability of the resistivity distribution over time, the average deviations between transimpedance measurements and simulations were less than 2%, and the maximum deviation was less than 5% over 7 days (Fig. 6c and d).

### EIT experiment results

**Figure 7:**
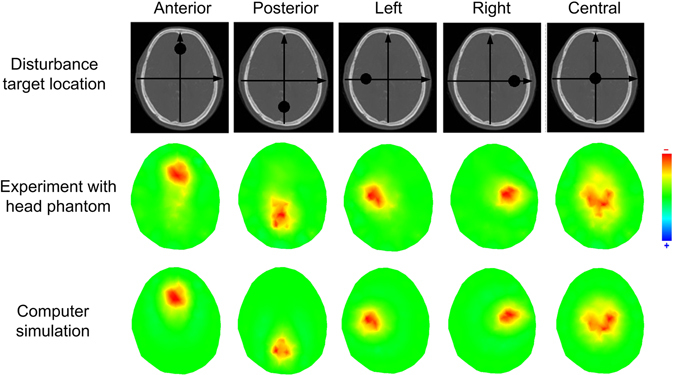
Comparison of electrical impedance imaging results. (**a**) Simulation results of imaging; (**b**) images from 3D-printed head phantom.

The brain EIT device was able to acquire data stably after the head phantom was connected. The imaging experiment was performed by adding perturbation in five reserved holes, separately. The imaging results were compared with the computer simulation, as shown in Fig. 7. The imaging results from the novel phantom for perturbation in different position is similar to computer simulation, including the location and shape of region of interest (ROI). This finding demonstrated the applicability and reliability of the novel head phantom for EIT experiments.

## Discussion

To provide a more accurate experimental platform for further studies of brain EIT, we created a novel 3D-printed head phantom with anatomically realistic geometry and continuously varying skull resistivity. The head phantom was favourably validated in terms of accuracy of resistivity distribution and geometry as well as EIT imaging.

In general, 3D printing techniques offer particular benefits to model the sophisticated geometry, especially for irregular anatomical structures in medical applications. Nevertheless, we must obtain a method of accurately controlling the resistivity of 3D printing materials before applying this technique to the construction of the EIT head phantom. In this study, as the conductive composite materials with ABS as the matrix are often used in 3D printing field and showed excellent electric properties^25, 26^, we attempted to alter the resistivity of ABS/CB composites according to real values of human head tissues by proportionally mixing two types of ABS/CB particles in which we actually adjusted the content of conductive agent. Similar to the relevant studies^27^, the result showed that the relationship between material proportion and the corresponding resistivity approximately appeared an exponential function. Based on the empirical formula, we finally achieve to obtain the 3D printing materials with target resistivity. Another merit of ABS/CB composites is that, since CB is less susceptible to oxidation than metal conductive particles^28^, the resistivity of the printed objects also exhibited better temporal stability as shown in this paper.

With the proposed resistivity control method, the 3D printing technique can provide a standardized, efficient and repeatable solution for precise fabrication of an EIT head phantom. In particular, we were not only able to accurately construct the trilayer skull structure using materials with desired resistivity that fundamentally defined the continuously varying skull resistivity but also precisely replicate the superficial sulci and gyri of brain. Moreover, given that the head phantom with anatomically realistic geometry and resistivity distribution is also needed in studied of brain function, such as transcranial direct current stimulation (TDCS)^29^ and electroencephalography (EEG)^30, 31^, and the proposed method can be rapidly reproduced using a similar 3D printer and ABS/CB conductive composites, this study provides an available method for fabricating a more accurate head phantom for brain function studies related to bioelectricity and bio-magnetism.

Researchers have built numerous head phantoms for EIT study in the past^6–8, 13^. The most realistic is a head phantom based on a plaster skull^14^. Compared with that head phantom, although the resistivity distribution of the plaster skull model was inhomogeneous among sections for skull components, the resistivity was constant across each section. In contrast, the resistivity distribution of 3D-printed skull model varies continuously with thickness or PTD, namely, the resistivity decreases with the increase of PTD, which is consistent with the live human skull^11^. On the other hand, a four-layer structure of the 3D-printed head phantom is more similar to the real human head than the three-layer structure, and the 3D-printed phantom contains a brain parenchyma model, which includes the structure of superficial sulci and gyri. Consequently, the novel head phantom possessed continuously varying skull resistivity distribution and a more accurate anatomically geometry, providing a more accurate platform for brain EIT research.

However, the proposed 3D printed EIT head phantom also needs further improvements. First, the resistivity of skull and brain parenchyma are represented as anisotropic, and this anisotropy has effects on brain EIT^32^. However, we ignored the resistivity anisotropy in our phantom for the sake of simplicity, which is a common practice. Second, the human brain parenchyma includes more complex components and structure. For example, the white matter only contains axons, whereas grey matters contain soma and axons. The ventricle exists in the centre of the parenchyma. However, we did not build these components separately. Third, some minor tissues for human head, such as meninges and blood vessels, are ignored. Fourth, despite of the complex structure and resistivity distribution of the suture, we did not accurately model the suture. In future studies, we may incorporate these properties into the construction of the head phantom. In addition, as the real human scalp is a soft solid object and the contact between the scalp and electrodes is an important point, we may introduce a soft conductive material for 3D printing instead of saline to fabricate a more realistic scalp tissue.

## Conclusion

In this study, a novel head phantom with anatomically realistic geometry and resistivity distribution for brain EIT was developed based on the 3D printing techniques. We especially fabricated the skull model with continuously varying resistivity by independently and simultaneously printing the trilayer skull structures using materials with distinct resistivity. Consequently, the new phantom not only had a precise geometry of the human head but also an accurate resistivity distribution similar to that of the real human head, suggesting that it is suitable for future studies in brain EIT. In addition, the proposed construction method in this paper may be easily adapted to other conditions for manufacturing head phantoms for brain function research, such as EEG and TDCS.

## Methods summary

### The method of accurately controlling the resistivity of materials for 3D printing

First, several types of high-quality commercial ABS/CB conductive composite particles were obtained and analysed with regards to resistivity properties. Next, we carefully selected two types with resistivity values that closely covered the range of head tissues. Finally, based on the two selected types of ABS/CB composite particles, we built a mathematical relationship between the mixture proportions of materials and the corresponding resistivity. According to the mathematical relationship, we finally achieved a synthetic resistivity-controllable 3D printing material by altering the proportion of those two types of ABS/CB particles.

### Fabrication of skull phantom with realistic inhomogeneous resistivity distribution

The skull model was created using the dual-nozzle 3D printer (Makerbot Replicator 2X, Makerbot, USA). One nozzle printed the shell (compact bone) with the material with a resistivity of 220 Ω·m, and the other nozzle simultaneously printed the infill (diploe) with the material with a resistivity of 20 Ω·m, in which the thickness of outer shell is fixed and inner fill varied with the thickness of the skull parts. Additionally, the total thickness of the two outer shells was 4.2 mm. Due to the large variation in thickness of the skull, when the thickness of skull section was less than 4.2 mm, the model was automatically printed as a single compact bone structure without the diploe layer, which was also similar to real skull tissue. Finally, the separated 3D-printed skull model sections were spliced together with conductive epoxy adhesive, whose resistivity is similar to the resistivity of the suture. After the skull model was printed, the resistivity distribution of the 3D-printed skull model was verified by direct comparison with resistivity measurements of live human skull.

### Construction of the entire novel head phantom and validation of the resistivity stability

After the fabrication of the skull model was completed, the brain parenchyma model was printed with material with the same resistivity as the real tissue, and the outer container structure for the entire head phantom was printed with non-conductive ABS. Then, we selected a NaCl solution with resistivity consistent with scalp or CSF to simulate the corresponding tissue. Finally, four tissue models (scalp, skull, CSF, and brain parenchyma) were combined to form a completed head phantom, and the stability of the phantom resistivity was verified by measuring its transimpedance.

### EIT Experiment based on the new 3D printed head phantom

The EIT experiment was performed to verify the applicability of the head phantom. The EIT imaging system was a high-precision electrical impedance apparatus from our research group^33^. Prior to the experiment, 10 ml 0.06% NaCl solution (the resistivity is 6.4 Ω·m at 25 °C, which is the same as that of the brain parenchyma model) was added to the five reserved holes in the brain parenchyma model. During the experiment, 10 ml 0.54% NaCl solution was added to the holes to simulate the cerebral haemorrhage as the concentration of the solution became 0.3% and resistivity became 1.64 Ω·m. Meanwhile, we conducted a computer simulation to verify the physical model experiment. In the computer simulation, the resistivity distribution and disturbance target is similar to that of the head phantom.

### Data Availability

All data generated or analysed during this study are included in this published article (and its Supplementary Information files).

## Electronic supplementary material

Full methods
