# Supplementary material for: A novel 3D-printed head phantom with anatomically realistic geometry and continuously varying skull resistivity distribution for electrical impedance tomography
Source: Sci Rep. 2017 Jul 4;7:4608. doi: 10.1038/s41598-017-05006-8 (PMC5496891; doi:10.1038/s41598-017-05006-8)
Supplement: Supplementary file 1 — Full methods [file 41598_2017_5006_MOESM1_ESM.pdf]

A novel 3D-printed head phantom with anatomically realistic geometry and continuously varying skull resistivity distribution for electrical impedance tomography

Jie Zhang[+], Bin Yang[+], Haoting Li, Feng Fu, Xuetao Shi, Xiuzhen Dong* and Meng Dai*

Faculty of Biomedical Engineering, Fourth Military Medical University, 169 West Changle Road, Xi′an 710032, China.
*Corresponding. dongxiuzhen@fmmu.edu.cn (X.D.); daimeng@fmmu.edu.cn (M.D.)
[+]These authors contributed equally to this work.

Full methods

1 The method of accurately controlling the resistivity of materials for 3D printing. Direct printing of the head tissues (i.e., skull and brain parenchyma in this paper) with the accurate resistivity necessitates an approach to control the resistivity of 3D printing materials. Traditionally, acrylonitrile butadiene styrene (ABS) is one of the most widely used materials in fused deposition modelling (FDM) 3D printing and carbon black (CB) is a conductive additive with excellent performance. Hence, ABS/CB conductive composites can theoretically be employed to build a model with a specific resistivity property as needed[1]. A direct method of obtaining resistivity-controllable 3D printing materials involves adding CB to ABS at different proportions, but professional instruments or sophisticated toxic agents are required to mix CB into ABS. These materials are currently inaccessible for our lab. Therefore, a reliable alternative method for controlling the resistivity of 3D printing material was developed. First, several types of high-quality commercial ABS/CB conductive composite particles were obtained and analysed with regards to resistivity properties. Next, we carefully selected two types whose resistivity values closely covered the range of head tissues. Finally, based on the two selected types of ABS/CB composite particles, we built a mathematical relationship between the mixture proportion of those particles and the corresponding resistivity. According to the relationship, we finally achieved a synthetic resistivity-controllable 3D printing material by altering the ratio of the two types of ABS/CB particles.

To analyse the resistivity properties of ABS/CB 3D-printed materials, we first established a method of resistivity measurement. At first, ABS/CB particles were fabricated into a filament with a diameter of approximately 1.75 mm using a single screw extruder in preparation for 3D printing (Fig. S1a)[2]. Second, cubic samples (20 mm x 20 mm x 20 mm) were printed by a commercial 3D printer (Makerbot Replicator 2X, Makerbot, USA) (Fig. S1b) using the prepared filaments for resistivity measurement. The resistivity measurement system included an impedance analyser (Solartron SI 1260, Schlumberger, USA) with a general-purpose interface board (GPIB) and a computer for system control and data input/output (Fig. S1c). The measurement pattern was based on the standard four-electrode method. The measuring cell is

presented in Fig. S1d, where the measuring electrodes were conductive rubber film with a resistivity less than 10^{-4} Ω·m. Using the elasticity of conductive rubber, full contact between the sample and the electrodes was achieved to reduce the contact resistance through a proper pressure. The sample was measured with 0.5 mA drive current in sweep frequency mode across the range of 1 Hz – 1 MHz. Each sample was measured thrice, and the average was then calculated to reduce measurement error. During measurement, the samples were placed into a temperature control box (Fig. S1c) to maintain them at room temperature (25.0 ± 0.5 °C).

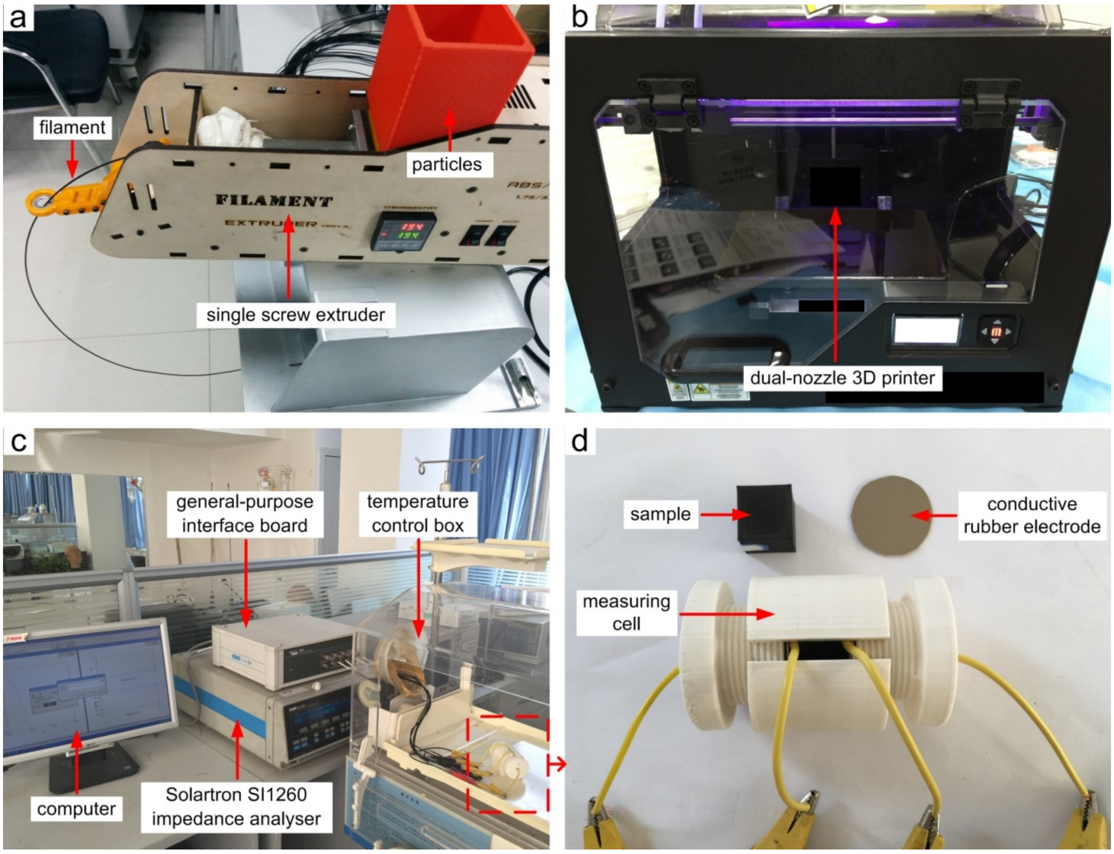

Figure S1. Material processing equipment and the resistivity-measuring device. (**a**) The single screw extruder for building ABS/CB particles into filaments; (**b**) the dual-nozzle 3D printer used for cubic sample preparation and model printing; (**c**) resistivity measuring device, including an impedance analyser, a general-purpose interface board (GPIB), a computer and a temperature control box; (**d**) measuring cell, including the conductive rubber electrode and the sample.

After careful investigation, we found that two types of ABS/CB particles with a volume fraction of 10% and 20% CB (referred to as ABS/CB$_{10\%}$ and ABS/CB$_{20\%}$, respectively) (Guangzhou Plastic Technology Co., Ltd., China) exhibited suitable resistivity properties for candidates. The resistivities of ABS/CB$_{10\%}$ and ABS/CB$_{20\%}$ were 426.6 ± 12.5 Ω·m and 6.4 ± 1.7 Ω·m at the frequency of 1 kHz, respectively, which generally covers the resistivity range of skull and brain parenchyma (Table S1). As shown in Fig. S2, the resistivities of the two ABS/CB printed objects exhibited stability over frequency (1 Hz - 1 MHz) and time domains (7 days).

Therefore, we tended to use ABS/CB$_{10\%}$ and ABS/CB$_{20\%}$ to build the mathematical relationship between the mixed proportion of those ABS/CB particles and the corresponding resistivities which were supposed to be in the range of skull and brain parenchyma (Table 1). ABS/CB$_{10\%}$ and ABS/CB$_{20\%}$ were

properly mixed with different proportions of ABS/$CB_{20\%}$ at 10% intervals, and then we printed six samples for each proportion to measure their resistivities. Finally, the mathematical model between proportions of ABS/$CB_{10\%}$ and sample resistivity was fitted. Theoretically, we were able to obtain an arbitrary resistivity within the range of skull and brain parenchyma.

Tissues	Resistivity (Ω·m)
Cerebrospinal Fluid (CSF)	0.56[15]
Scalp	2.27[16]
Brain parenchyma	6.67[17]
Skull	79.43–265.46[3]

Table S1. Resistivities of head tissues at a frequency of 1 kHz

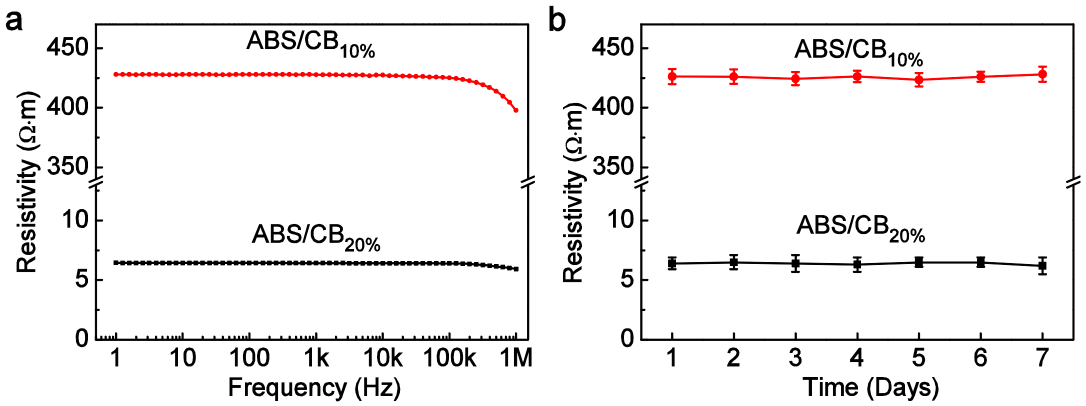

Figure S2. The resistivities of ABS/$CB_{10\%}$ and ABS/$CB_{20\%}$ composites. (**a**) Resistivity spectra of the two materials in the frequency of 1 Hz – 1 MHz; (**b**) resistivity measurements as a function of time over 7 days. The average error is less than 3% (Frequency = 1 kHz).

2 Fabrication of a skull phantom with realistic inhomogeneity resistivity distribution. The typical skull structure consists of three layers: top compact bone, diploe, and low compact bone. The non-uniformity of the skull thickness was mainly due to the variation of inner diploe thickness, and the thicknesses of the two outer compact bone layers were relatively constant[3,4]. Accordingly, we first reconstructed an accurate computer-aided design (CAD) model of a human skull using CT image sequences and divided the CAD model into eight skull sections (one frontal bone, two sphenoid wing bones, two temporal bones, two parietal bones and one occipital bone) as shown in Fig. S3a[5]. Second, before printing, the separated CAD model was sliced to a structure similar to skull anatomy. The model was mainly composed of a trilayer structure, and the structure varied in thickness. Specifically, the model had two outer compact bones and one inner diploe if the total thickness was greater than 4.2 mm given that a single-layer compact bone is approximately 2.1 mm[6]. When the total thickness was less than or close to 4.2 mm, we modelled the skull as a pure compact bone. According to the differences of the interior composition and structure, the skulls could be classified into 4 types: standard compact skull, quasi-compact skull, quasi-trilayer skull and standard trilayer skull[3] (Fig. S3). Third, the physical models of skull sections were individually created using the dual-nozzle 3D printer in which we set the raster

width to 0.7 mm and the perimeter number of vertical shell to three. Based on the proposed method of controlling the resistivity of 3D printing materials, one nozzle printed the shell (compact bone) using the material with a resistivity of 220 Ω·m, and the other nozzle simultaneously printed the infill (diploe) using the material with a resistivity of 20 Ω·m[7]. Finally, the separated 3D-printed skull model sections were spliced together with conductive epoxy adhesive with resistivity similar to the resistivity of the suture to form a completed skull phantom.

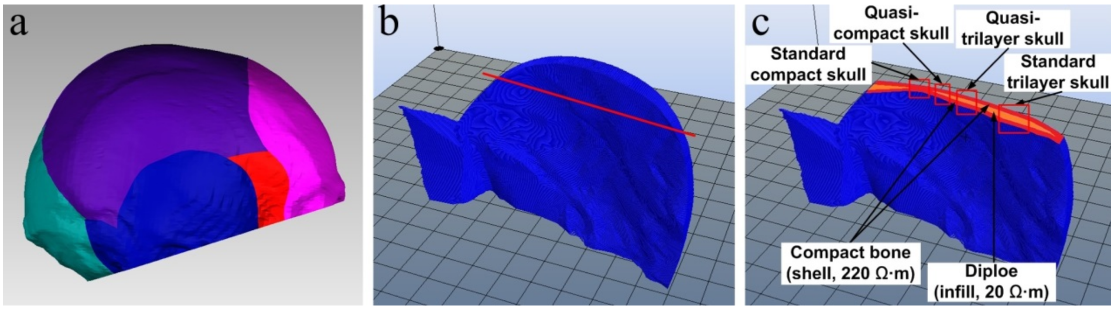

Figure S3. The CAD model of skull and a structure of left parietal bone that is similar to skull anatomy by slicing. (**a**) The CAD model of skull by medical imaging and 3D reconstruction; (**b** and **c**) the structure of left parietal bone that is similar to skull anatomy by slicing. The cross section revealed the definition of diploe (orange) and compact bone (red). Based on different structures of the skull, the model was classified into standard compact skull, quasi-compact skull, quasi-trilayer skull and standard trilayer skull.

After the fabrication of skull phantom was complete, we verified its performance with respect to resistivity distribution and geometric accuracy compared with the real human skull. The resistivity of human skull has been studied by numerous researchers[3,4,6,8-10], and the latest study was performed by Tang et al from our group[3]. In that study, 388 plugs (14 mm diameter) were excised from 48 skull flaps of patients undergoing neuro-surgery and classified into six categories (including the abovementioned four types and the other two ignored for simplification in this study). Then, resistivity was carefully measured. Therefore, in this study, we also cut out plugs of the same size from the 3D-printed skull model, categorized them into those four types and measured their resistivity. Finally, a comparison of resistivity between the proposed skull phantom and the live human skull was performed. Specifically, the two-sample t test was applied to test the difference in resistivity for each skull category, and p-values more than 0.05 were considered to be no statistically significant. On the other hand, the relationship between the resistivity of trilayer skull phantom and the percentage on thickness of diploe (PTD) was evaluated.

Regarding geometric accuracy, based on the dimensional analysis of FDM fabricated medical replicas by El-Katatny et al[11], 15 landmarks on the left parietal bone and the entire skull were selected for linear measurement analysis. Table S2 presents the linear measurement definition of these landmarks. Fig. S4 indicates the physical location of these landmarks. For each landmark, an average of six measurements was calculated, and the difference between measurements on the CAD model and the 3D-printed model are presented in terms of mm and percentages.

Landmark name	Definition
AB	The linear distance between the intersection of the sagittal and coronal sutures (A) and the intersection of the coronal and parietotemporal sutures (B)
BC	The linear distance between the intersection of the coronal and parietotemporal sutures (B) and the intersection of the parietotemporal and lambdoidal sutures (C)
CD	The linear distance between the intersection of the parietotemporal and lambdoidal sutures (C) and the intersection of the lambdoidal and sagittal sutures (D)
AD	The linear distance between the intersection of the sagittal and coronal sutures (A) and the intersection of the lambdoidal and sagittal sutures (D)
T1	The thickness of midpoint of AB
T2	The thickness of midpoint of BC
T3	The thickness of midpoint of CD
T4	The thickness of midpoint of AD
H1	The distance from the highest point of the coronal suture to the reference plane
H2	The distance from the highest point of the parietotemporal suture to the reference plane
H3	The distance from the highest point of the left parietal to the reference plane
H4	The distance from the highest point of lambdoidal suture to the reference plane
SL	The distance between the external occipital protuberance and the frontal bone
SW	The distance between left and right temporal bone
SH	The maximum distance between the top and bottom of the skull model

Table S2. Landmarks definitions used for the linear measurement

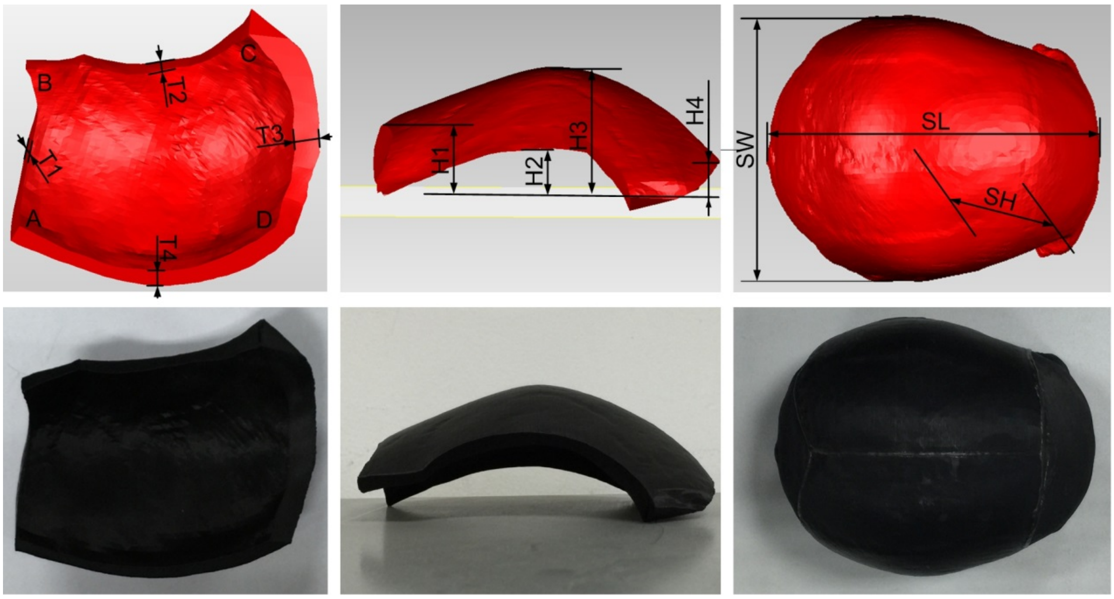

Figure S4. Linear measurements of landmarks used in the comparison between the CAD model and 3D-printed model

3 Construction of the entire novel head phantom and validation of the resistivity stability.

After the fabrication of the skull model was completed, the brain parenchyma model was printed with material exhibiting the same resistivity as the human tissue, and the outer container structure for the entire head phantom was printed with the non-conductive ABS. Afterwards, we selected a NaCl solution with resistivity consistent with scalp or CSF to simulate the corresponding tissue. Finally, four tissue models (scalp, skull, CSF, and brain parenchyma) were combined to form a completed head phantom, and the stability of the phantom resistivity was verified by measuring the transimpedance.

First, the CAD model of the brain parenchyma was obtained. The method was similar to that used for the skull CAD model. During the establishment of the CAD model, five cylindrical holes with a diameter of 20 mm and a depth of 50 mm were added to the brain parenchyma model at different locations (anterior, posterior, left, right, and central) for the subsequent EIT imaging experiments. The CAD model of brain parenchyma consisted of six divided sections (left and right frontal lobes, left and right parietal lobes, and left and right occipital lobes). Afterwards, the printing and splicing of the brain parenchyma model was performed. The printed material possessed a resistivity consistent with brain parenchyma (Table S1) that was obtained from the aforementioned resistivity control method. The material used for splicing was a conductive epoxy adhesive with the same resistivity of the brain parenchyma.

Regarding the container structure, the outer surface of the CAD model of the skull was extracted and expanded 5 mm outwards followed by another outward extension to form an outer container with a thickness of 5 mm. Then, two layers of small holes, 16 in each layer, were uniformly surrounded on the container model for the addition of electrodes. After the outer container was printed and spliced, the Ag/AgCl electrodes were added at the reserved small hole.

The completed models of different layers were then combined to form a four-layer head phantom. First, to fix the brain parenchyma model to the inside of the skull model, three small cylinders were attached to the inner surface of the skull. The height of small cylinder is determined by the distance between the corresponding position of the skull and the brain parenchyma. Similarly, the small cylinder was added to the inner surface of the outer container to fix the skull. Second, after the assembling of the brain parenchyma, the skull model and the outer container, a NaCl solution (concentration 0.9%, resistivity 0.56 Ω·m) with resistivity similar to the CSF (Table S1) was added to the space between the brain parenchyma and the skull to simulate CSF. A NaCl solution (concentration 0.2%, resistivity 2.27 Ω·m) with resistivity similar to the scalp (Table S1) was added to the space between the outer container and the skull to simulate the scalp, forming a four-layer structure of the head phantom. Finally, the supporting structure for the head phantom was assembled to form an integrated EIT experimental platform.

To assess the stability of the entire head phantom, transimpedances were measured, and the results were compared with simulation values as reported previously[5,12]. The current was input from electrodes 1 and 9, and the voltages of other adjacent electrode were measured with the impedance analyser (Fig. S5a). The transimpedance was defined as the ratio between the measured voltage and the input current. The input current was 1.25 mA, and the frequency was 1 Hz – 1 MHz. To evaluate the measured value through comparisons, the transimpedance was simulated using finite element simulation software (COMSOL Multiphysics 5.2, COMSOL Inc., Sweden). The structure of the finite element simulation model was a two-dimensional cross-section of the electrode plane of the new head phantom, as shown in Fig. S5b. The resistivity settings for the different layers were the same as those for the head phantom. In addition, to evaluate the resistivity stability of the head phantom over time, we measured the transimpedances every day within seven days at the same ambient temperature and compared the

results with simulation.

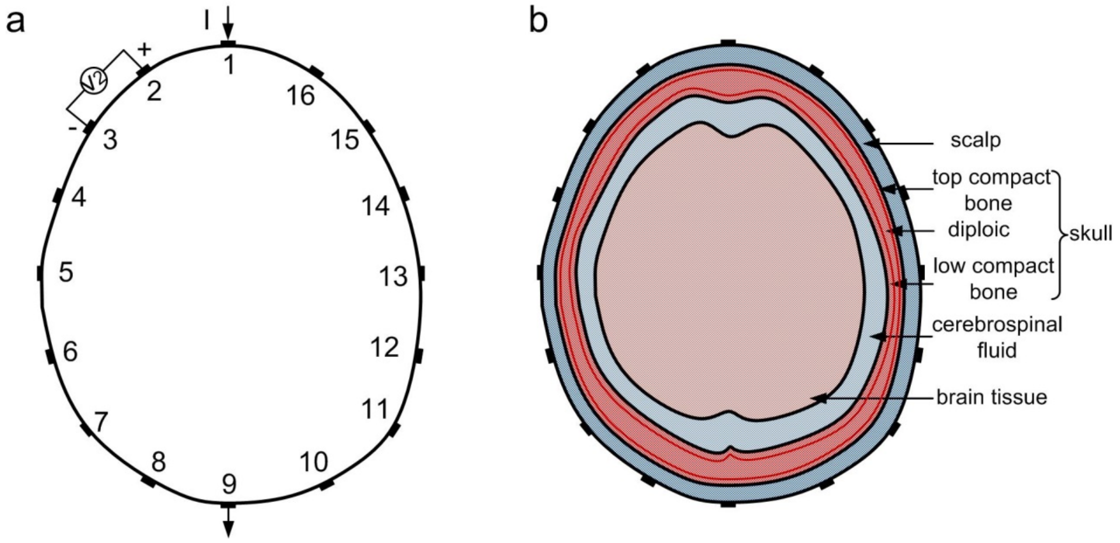

Figure S5. Measurement of transimpedance. (**a**) The current was input from electrodes 1 and 9, and the voltages between the remaining adjacent electrodes were measured. The number of measurement results is indicated with the smaller electrode number; (**b**) layered structure of 2D simulation model for transimpedance calculation.

4 EIT Experiment based on the new 3D printed head phantom. The EIT experiment was performed on the experiment platform to verify the applicability and reliability of the platform. The EIT imaging system was a high-precision electrical impedance apparatus from our research group[13]. Prior to the experiment, 10 ml 0.06% NaCl solution (the resistivity is 6.4 Ω·m at room temperature, which is the same as that of the brain parenchyma model) was added to the hole in the brain parenchyma model. During the experiment, 10 ml 0.54% NaCl solution was added to the reserved holes to simulate the cerebral haemorrhage as the concentration of the solution became 0.3% and resistivity became 1.64 Ω·m. Meanwhile, we conducted a simulation experiment using the Simulation and Experimentation Platform for EIT (EitSep)[14]. The computer simulation model was incorporated with the inhomogeneous skull resistivity and four-layer head structure, which is similar to the novel head phantom.

Reference

1. Ou, R., Gerhardt, R. A., Marrett, C., Moulart, A. & Colton, J. S. Assessment of percolation and homogeneity in ABS/carbon black composites by electrical measurements. *Composites Part B Engineering* **34**, 607-614 (2003).
2. Baechler, C., Devuono, M. & Pearce, J. M. Distributed recycling of waste polymer into RepRap feedstock. *Rapid Prototyping Journal* **volume 19**, 118-125 (2012).
3. Tang, C. *et al.* Correlation between structure and resistivity variations of the live human skull. *Biomedical Engineering, IEEE Transactions on* **55**, 2286-2292 (2008).
4. Law, S. K. Thickness and resistivity variations over the upper surface of the human skull. *Brain Topography* **6**, 99-109 (1993).
5. Li, J.-B. *et al.* A new head phantom with realistic shape and spatially varying skull resistivity distribution. *Biomedical Engineering, IEEE Transactions on* **61**, 254-263 (2014).
6. Akhtari, M. *et al.* Conductivities of three-layer live human skull. *Brain topography* **14**, 151-167 (2002).
7. Tang, C. *Studies on impedance spectroscopy characteristics and inhomogeneous resistivity distribution of the live human skull*, Fourth Military Medical University, (2008).

8. Akhtari, M. *et al.* Conductivities of three-layer human skull. *Brain Topography* **13**, 29 (2000).
9. Oostendorp, T. F., Delbeke, J. & Stegeman, D. F. The conductivity of the human skull: results of in vivo and in vitro measurements. *IEEE Transactions on Biomedical Engineering* **47**, 1487 (2000).
10. Hoekema, R. *et al.* Measurement of the conductivity of skull, temporarily removed during epilepsy surgery. *Brain Topography* **16**, 29-38 (2003).
11. El-Katatny, I., Masood, S. H. & Morsi, Y. S. Error analysis of FDM fabricated medical replicas. *Rapid Prototyping Journal* **16**, 36-43 (2010).
12. Sperandio, M., Guermandi, M. & Guerrieri, R. A four-shell diffusion phantom of the head for electrical impedance tomography. *Biomedical Engineering, IEEE Transactions on* **59**, 383-389 (2012).
13. Shi, X., You, F., Fu, F., Liu, R. & Dong, X. in *2005 IEEE Engineering in Medicine and Biology 27th Annual Conference.* 1492-1495.
14. Yang, B. *Research on Image Reconstuction Incorporating with the Inhomogeneous Distribution of the Skull Resistivity in Electrical Impedance Tomography for the Brain*, Fourth Military Medical University, (2013).
15. Baumann, S. B., Wozny, D. R., Kelly, S. K. & Meno, F. M. The electrical conductivity of human cerebrospinal fluid at body temperature. *IEEE Transactions on Biomedical Engineering* **44**, 220-223 (1997).
16. Burger, H. C. & Milaan, J. B. V. Measurements of the specific Resistance of the human Body to direct Current. *Journal of Internal Medicine* **114**, 584–607 (1943).
17. Geddes, L. A. & Baker, L. E. The specific resistance of biological material—A compendium of data for the biomedical engineer and physiologist. *Medical & Biological Engineering* **5**, 271-293 (1967).
